# How to succeed at medical interviews

**DOI:** 10.1097/IJ9.0000000000000021

**Published:** 2017-06-15

**Authors:** Kiron Koshy, Katharine Whitehurst, Alison Liu, Buket Gundogan, Alexander Fowler, Riaz Agha

**Affiliations:** a Brighton and Sussex University Hospital; b Royal Devon and Exeter Hospital; c UCL Medical School, University College London; d Guy's and St. Thomas' NHS Foundation Trust, London, UK

**Keywords:** Interview, Application, Portfolio, Medical interview, Core surgical training, Core medical training, Specialty application, Medical school

## Abstract

Few people truly relish being interviewed. But as a professional, you will go through a number of interviews in your life that will affect your career progression. In this article, we provide some tips on how best to prepare for a medical interview, both in terms of your portfolio and interview practice. Advice is also given on how to maximize performance during the interview and gain the most from reflection afterwards.

Few people truly relish being interviewed. But as a professional, you will go through a number of interviews in your life that will affect your career progression. This is not just in relation to job advancement, but also for supervisor meetings and medical review of competence/revalidation^1^,^2^.

Interview skills also overlap with presentation skills and examination viva skills, in that all three skills are “performing in front of others under pressure” and each involves some degree of nerves. Preparation for interviews and building interview skills will, therefore, go a long way toward future career success^3^,^4^. To tackle this subject, interviews can be divided into three stages: before, during, and after.

## Before the interview

The aim of your preparation should be: “to learn how to sell yourself in a positive light in relation to the post without sounding arrogant.” The best way to achieve this elusive balance is to:

### 1. Know yourself

To be an effective team member a person needs to know themselves well—their personality, strengths and weaknesses, values, motivations, experience, career aims, and skills^5^,^6^. People who do not have insight into these features have a tendency to answer questions in an arrogant manner, or grossly undersell themselves. It is worth having a think about the following questions:

- What are your weaknesses/strengths?
- How would you describe yourself?
- How would your colleagues and friends describe you?

Also think about specific achievements and examples. It is important to explore what was achieved and what was your contribution^7^:

- What is your best research project?
- What is your best audit project?
- Where have you shown great leadership and management skills in your career?
- Where have you shown great teaching skills?
- What is your biggest strength/weakness.

The STARR acronym is very helpful in framing your answer^4^.

**Situation:** describe briefly the situation that you were in, to provide some context for the interviewer.

**Task:** What were the goals that you/your team were trying to achieve?

**Action:** What were your individual actions toward achieving this goal?

**Result:** describe the outcome in this scenario and the implications of your work.

**Reflection:** What was done well and what could have been done better?

In providing an answer, the “action” and “reflection” components should reserve the most time and emphasis. Bear in mind that the action should describe only your role in this scenario. Try to use the word “I,” rather than “we” as the latter may give the impression that you contributed less toward the overall result^3^.

### 2. Keep a good portfolio

This is an often under-appreciated area by applicants. Most interviews beyond medical foundation applications will require you to bring a portfolio of your achievements to the interview. What to include and how to structure this is often provided on the organization’s website^8^.

Prepare your portfolio as early as you can. As you write a list of your achievements, you will inevitably come across accomplishments for which you have no physical proof. Allowing yourself time to obtain/find certificates will save a lot of stress before the interview^9^.

Interviewers may look at your portfolio before the interview, but you will also often have your folder in front of you in a portfolio station of the interview. As a result, keeping an organized portfolio and being able to flick quickly to the relevant page presents a good image to the interviewer. Many interviews also have a significant portion of marks available for how organized and esthetic your portfolio is. Keeping it succinct and intuitively laid out, therefore, scores you points both directly and indirectly. Buying a professional folder with colored coded dividers is a good investment^10^.

### 3. Know the Job

For many medical jobs (especially academic foundation/specialty training posts) there are clear person specifications and requirements matrices available online. Therefore, it is reasonable to anticipate questions alongside the characteristics employers desire^11^,^12^. For example, if they want people who are future leaders, a reasonable question would be “tell us what makes you a good leader?”

There are many other ways to find out more about a post:

- Speak to a current post holder or someone holding a similar post.
- Check the organization’s website.
- Speak to the department/job lead.
- Speak to the departmental secretary.
- Speak to the regional adviser in the specialty where possible.
- Speak to the HR department and ask for a copy of the annual report.
- Obtain and read carefully the job description, person specification, and all other data that may be sent to you.

Do this comprehensive research and you will start to see which parts of your past are particularly relevant to the post and how you can specifically contribute to the department.

### 4. Anticipate

It is a great idea to keep an ever-expanding list of interview questions, from reading the medical press, asking those who have just been to an interview, your own interviews, and asking those on interview panels which questions tend to be answered badly^13^. Many academic and specialty interviews include a research component where you may be asked to discuss an abstract provided to you on the interview day. Abstracts selected often come from high-impact journals like the *New England Journal of Medicine* and The *Lancet*. Regularly searching these journals, will help you become more familiar with the way data are represented in these abstracts^14^.

### 5. Articulate fluently

#### Preparation

Not everyone can think fast off the top of their head under pressure. Many feel that a rehearsed answer can sound robotic. However, in professional interviews there are often topics that interviewers will want to hear about, be it for management options in a clinical scenario, or skills that you have acquired in your work. There will also be experiences that you will want to include; preparing and rehearsing answers makes sure you will not forget about these^15^.

#### Rehearse

A good approach to answering potential interview questions during preparation is to write the answer down rather than trying to keep it in your head. Once your answer is in front of you, it is easier to analyze, refine, and seek feedback from others.

Practicing interviews by speaking out loud allows you to get used to talking about yourself in positive terms. Communicating clearly and confidently in interview is an essential skill^16^.

Gaining some feedback from others (colleagues, family, friends, and tutors) in mock interviews can help you to start finding some effective phrases. Doctors are notoriously bad at or uncomfortable with “selling themselves”. However, interviews are simply an occasion for your future team members to gain some insight into your character and your accomplishments. Practice to become well versed in talking about your key selling points with ease.

## On the day of the interview

### Be prepared

Preparation is key, get a good night’s sleep, know your CV, look presentable, and arrive early. Remember any documents you were asked to bring^17^.

### Communication skills

#### Body language

Look relaxed, do not overdo hand movements, remember to smile, and make plenty of eye contact. Non-verbal cues are vital in building rapport with the interviewer^18^.

#### Answering questions

- Take a deep breath and ensure the structure of your answer includes: (1) statement, (2) evidence/example, (3) summarize with explicit answer to the questions.
- Keep your answers open and balanced.
- Begin with a structure to what you want to say and then follow through with the details.
- The ability to know when to stop talking is important—If you feel you have answered a question fully, stop, even if there is a brief period of uncomfortable silence.
- If you feel your last answer was poor, put it behind you and focus on the next question, you may be able to redeem yourself. This is especially important in a station-based format with rest stations, as you should use these to reset and prepare for the next station, rather than analyzing your recent failures^17^.
- Try and come across as the sort of person the interviewers would like to work with. Generally this means being bright, stable and controlled, with good interpersonal and leadership skills, while being reasonably relaxed, conscientious, and warm-hearted^19^.

#### Listen

Many candidates do not answer the question they have just been asked. Listen very carefully to the question and do not rush into an answer. If a question is not clear in its remit, then ask for some clarification^20^.

## After the interview

Many people breathe a sigh of relief as they walk out the door of the interview room and instantly forget everything that took place. This is a mistake, as reviewing and reflecting is an invaluable learning opportunity for improving your approach at the next interview^21^.

### Review

Jot down as many questions as you can recall and also the answers you gave. Writing them down immediately will improve your recall of the details, but also of your thought processes and emotions at the time. Try to write an honest appraisal of yourself in this interview and ask yourself:

- Which questions went well or badly?
- You will have prepared for some of these questions before the interview, but how did you do under pressure?
- Did you feel that each answer was received well or did they seem to be looking for something additional?
- Did the interview have a relaxed happy feel to it or did you sense tension or hostility?
- Did you do or say anything that you would try to repeat or avoid in future interviews?
- How did the interview begin and end? Was each satisfactory?

Reviewing these questions will make sure you do not fall into the same traps and build on things that you did well^22^.

### Get feedback

If you did not get the job, it is vital to attempt to gain interview feedback from the panel. Obtaining feedback can sometimes be difficult. Increase your chances by:

- Trying to phrase your request for feedback in a constructive and positive way for yourself. If you enjoyed your interview—say so.
- Try to gain useful feedback from comments like “you did really well—but there was somebody just a bit more suitable” by replying, “I wondered if you could point out any areas where I could do better at interview or any areas in my career where I should pay more attention.” Try to do this as soon as possible after you receive a rejection—their recall will not last long.
- Even if the feedback suggests that they think you are unlikely to succeed in this career route with your given skills and experience—this can be invaluable by either stiffening resolve, or helping one to come to terms with the need to seek some objective career guidance and look at a wider range of options^4^.

## Getting better at interviews

If you feel like you need further practice, there are a number of ways to approach this^5^,^6^. These include:

- Public speaking training and practice.
- Selling skills training (so that you can adopt a more balanced approach to selling yourself if you are not that comfortable with doing this).
- Seeking interview training.
- Get friends and colleagues to interview or video you and ask them to be brutal.

## Summary

- Interviews and your performance in them can make or break a career pathway.
- Developing a strategy for doing well in them is a sensible investment—preparation is key!
- Try to learn from failure by reviewing your performance and gaining feedback.
